# Generalized focused-ion-beam milling strategy to tune mechanical properties of AFM cantilevers for single-molecule force spectroscopy studies

**DOI:** 10.1063/5.0257032

**Published:** 2025-06-13

**Authors:** Christopher B. Hatchell, David R. Jacobson

**Affiliations:** Department of Chemistry, Clemson University, Clemson, South Carolina 29634, USA

## Abstract

Atomic force microscopy (AFM)-based single-molecule force spectroscopy (SMFS) enables the characterization of individual biological molecules through the application of mechanical force. The spatiotemporal resolution of such measurements depends greatly on the AFM cantilever that is used, specifically its stiffness, hydrodynamic drag, and material composition. Prior work has shown that focused ion beam (FIB) lithographic modification of small cantilevers can be used to lower the spring constant (and thus force noise) and drift while maintaining a relatively fast time response. Published methods for implementing such optimization rely on specific FIB instruments and cantilever types, limiting broad implementation of these methods to improve SMFS data quality. Here, we show that it is possible to achieve such optimized properties using generalized techniques applicable to a broader array of FIB instruments and starting from new types of cantilevers that are presently commercially available. Modified cantilevers exhibited a 90% reduction in spring constant, sub-pN force drift to tens of seconds, and a time response of ∼25 *μ*s in the liquid environment relevant to biological measurements.

## I. INTRODUCTION

**FIG. 1.**
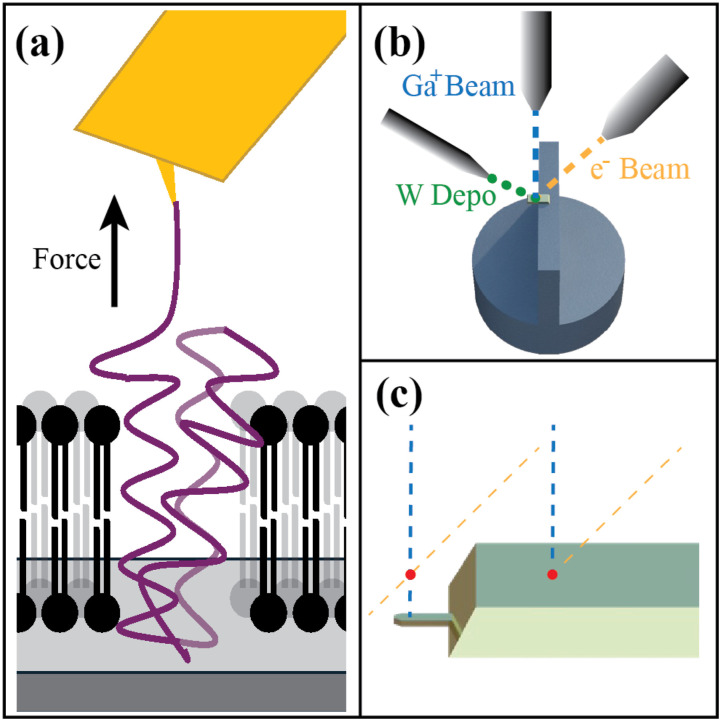
(a) Typical AFM-based SMFS experiment to unfold a membrane protein. (b) Schematic of cantilever and beam setup including tungsten deposition spray (*green*), Ga ion beam (*blue*), and electron beam (*yellow*). (c) Location of eucentric point (*red dot*) on top of the chip vs over the cantilever (not drawn to scale).

Single-molecule force spectroscopy (SMFS) uses mechanical forces to probe individual molecules and their interactions,^1^ including ligand-receptor interactions,^2^ nucleic acid unzipping,^3,4^ and protein unfolding.^5–10^ Understanding the folding of proteins is imperative for determining structure and function. Atomic force microscopy (AFM)-based SMFS can be used to identify intermediates in the mechanical unfolding pathway of proteins and to characterize the equilibrium thermodynamics of these intermediates.^5,6,11,12^ In an AFM-based SMFS experiment, one end of the protein under study is attached to the cantilever, and the other end is anchored to a surface via flexible linkers (in the case of soluble proteins) or embedded in a lipid bilayer [in the case of membrane proteins, Fig. 1(a)]. The cantilever is then pulled away from the surface, raising the tension felt by the protein. When the protein undergoes a conformational change, the newly unfolded amino acids add to the linker contour length, reducing the tension and leading to cantilever relaxation toward its equilibrium position. Applied force is read out as a function of time by using a laser reflected off the back of the cantilever to report on its deflection, which is related to force via a calibrated spring constant.

Data quality in AFM-based SMFS studies—and the ability to resolve short-lived and closely spaced unfolding intermediates of proteins—is thus closely tied to the mechanical properties of the cantilever, especially its stiffness, hydrodynamic drag, and material composition (including metallic coatings). The stiffness is expressed in terms of the spring constant (*k*_*s*_) of the cantilever. Values of *k*_*s*_ in biological SMFS applications can be as low as ∼5 pN/nm, which is achieved using a relatively long (100 *μ*m) cantilever.^13^ Such stiffness is vastly lower than that used in air or vacuum tapping-mode imaging applications, which can be on the scale of 100 nN/nm.^14^ Cantilevers with lower *k*_*s*_ exhibit less thermal force noise. This is seen by setting the thermal energy in the vibrational degree of freedom (*k*_*B*_*T*/2, from the equipartition theorem) equal to the Hooke’s-law energy $\frac{1}{2}\frac{F^{2}}{k_{s}}$ in terms of force (*F*). Thus, *F*^2^ = *k*_*s*_*k*_*B*_*T* and $\delta F∼\sqrt{k_{s}}$.^15^ Therefore, a lower spring constant improves SMFS data quality by lowering thermal noise as it relates to the force. The second key mechanical property, time response (τ), is the cantilever’s time constant of exponential relaxation to equilibrium when *F* changes, such as when the molecule under study undergoes a conformational change. Especially in the liquid environment of biological measurements, τ scales with cantilever hydrodynamic drag. Values of τ as low as ∼1 *μ*s can be achieved using cantilevers with ultrashort dimensions (*L* = 9 *μ*m) and thus low drag, although these have stiffnesses on the scale of ∼100 pN/nm.^5,8^ By contrast, the long, soft cantilevers mentioned above have time responses of ∼500 *μ*s.^13^ A lower τ value improves SMFS data quality by allowing the cantilever to report on rapid conformational changes with higher fidelity, although lower τ typically comes at the cost of higher *k*_*s*_ and *δF*. Finally, prior work has shown that the gold and chromium coating applied to the silicon-nitride cantilever to reflect the detection laser causes drift in deflection and, therefore, in *F* over time.^16,17^ Thus, there is similarly a trade-off between providing a sufficiently reflective surface to achieve good detection signal-to-noise and removing the reflective coating to reduce drift.

Because of the trade-offs that historically exist between these parameters, there is great interest in methods that can simultaneously modify cantilevers to decrease the spring constant, drag, and drift. Prior work has shown success in working with cantilevers having a small cross-sectional area (and thus drag) and then both (i) modifying the geometry to lower *k*_*s*_^18–22^ (and further lower drag) and (ii) partially etching the Au/Cr surface coating to decrease drift.^16,19,23^ Both advancements were made using focused ion beam (FIB) lithography in conjunction with chemical etchants. In particular, the Ga-ion beam of the FIB was used to narrow the shaft of the cantilever (or divide it into two separate, small legs) in both plan and thickness dimensions. Beam theory predicts that a cantilever’s stiffness will decrease linearly with plan width and cubically with thickness. Indeed, the application of this method resulted in a ≳10-fold reduction in *k*_*s*_ when applied to both short (*L* = 38 *μ*m) and ultrashort (*L* = 9 *μ*m) cantilevers.^13,18^ In addition, the electron beam of the FIB instrument was used to deposit a protective patch of insulating material that was subsequently used as a resist when the Au/Cr layer was chemically etched away. The resulting gold patch preserved laser reflectivity while reducing cantilever drift. Cantilevers treated in this way demonstrated sub-pN force stability to ∼100 s in the case of short cantilevers and ∼10 s in the case of ultrashort cantilevers.^13,19^ The combined ability to resolve short-lived conformational states (facilitated by small τ) and to collect single-molecule data for extended periods (facilitated by low drift) has enabled, for example, the first single-molecule measurement of equilibrium energetics in a membrane protein.^6^

Despite their success in simultaneously improving time response, lowering force noise, and reducing drift, published cantilever FIB-modification methods are narrowly tailored to specific FIB instruments and types of cantilevers. Published methods rely on a FIB instrument with a focal range large enough to encompass both the cantilever and the plane of the top of the chip to which it is mounted (Δ*h* = 330 *μ*m), the ability to controllably defocus the beam to a specified blur diameter, and the ability to deposit transparent resistive material (tetraethyl orthosilicate, TEOS). Such capabilities are not universal across FIB instruments and limit the applicability of these methods. In addition, published methods modified specific types of commercially available cantilevers: the Olympus AC40TS (*L* = 38 *μ*m) and the Olympus AC10DS (*L* = 9 *μ*m).^13,18,19^ Both cantilevers have been discontinued by the manufacturer, severely limiting the applicability of the published methods that rely upon them.

Here, we show that modified techniques, applicable to a broader range of FIB instrumentation can reproduce previously reported cantilever performance improvements for the Olympus AC40TS BioLever Mini cantilever and, further, that these methods can be extended to a presently commercially available cantilever (Bruker PEAKFORCE-HIRS-F-B). We demonstrate the success of these modified techniques through mechanical and noise characterization in the liquid environment relevant to biological measurements. Extending FIB-modification methods to a broader range of instrument and cantilever types facilitates more widespread access to high-time-resolution AFM-based SMFS measurements that can reveal otherwise hidden protein unfolding intermediates and their corresponding energetics.

## II. TECHNICAL ADAPTATIONS FOR PLATFORM INDEPENDENCE

Adapting FIB modification techniques for broad instrumental compatibility requires addressing several key technical challenges that arise from differences in FIB system capabilities. Previous protocols relied on specific features—such as large focal ranges, precise beam defocusing control, and TEOS deposition—that are not universally available across FIB instruments. Here, we perform FIB-based modification of cantilevers using a Hitachi NB5000 instrument to demonstrate platform-independent alternatives to these procedures.

### A. Sample height and clearance

Previously published methods can monitor the sample by scanning electron microscopy (SEM) during FIB milling because the sample is positioned at the eucentric height, the specific location where the FIB and SEM beams intersect [Fig. 1(b)]. The NB5000 is designed for flat surfaces, assumes that the highest point of the sample is what will be imaged, and cannot accommodate higher samples because of tight clearance. Prior to loading the sample, a benchtop laser range finder is used to set the sample height such that the highest point is at the eucentric height. In the case of an AFM cantilever, this is the top surface of the chip to which the cantilever is attached, 330 *μ*m higher than the cantilever itself. Thus, once the sample is loaded, the FIB/SEM beam eucentric point is located at the top of the chip rather than the cantilever [Fig. 1(c)]. FIB focusing and milling are still possible below the eucentric height, but the sample will not be in the SEM field of view. We found that the progress of FIB milling can still be monitored by the SEM in a non-real-time manner by translating the sample stage horizontally to place the cantilever in either the Ga-ion or electron beam path.

### B. Defocusing FIB beam to thin cantilever

Prior literature uses a change in “blur diameter” to thin the cantilever by ablating material without cutting through it.^19^ While the ability to specify such a defocusing radius is not a capability of all FIB instruments, the same effect can be achieved by manually putting the instrument slightly out of focus. Although effective, this approach raises concerns about repeatability. We found that we could overcome this concern by specifying a certain change in voltage to the Ga-beam focusing lens. Furthermore, a comprehensive table of relevant cantilever properties for multiple different cantilevers is shown in the supplementary material, Table S1. The appropriate voltage change can be calibrated by defocusing by eye, measuring the width of material milled away from a test region of the chip, and adjusting as needed to be wide enough to encompass the region to be thinned. On the Hitachi NB5000 and for the cantilever geometries considered here, a ∼0.150 keV change sufficiently defocused the beam.

### C. Etching resist replacement

In published protocols, TEOS is deposited as a resist to allow a patch of reflective gold to be retained while removing most of the cantilever’s gold and chromium coating to lower cantilever drift.^13,16^ However, TEOS deposition is not a universal feature of FIB instruments. As an alternative, some FIB instruments are fitted to deposit high-purity tungsten due to its usefulness as a thin film in semiconductor devices.^24^ The tungsten is introduced via a gas nozzle [Fig. 1(b)] as gaseous tungsten hexacarbonyl [W(CO)_6_], which is then deposited as pure tungsten upon interaction with the Ga beam. TEOS, by contrast, is deposited using the electron beam. After serving as an etching resist for the gold and chromium, the tungsten is initially reflective and so could be left on the cantilever. However, exposure to salt-containing solutions relevant to biological measurements causes the tungsten to darken, leading us to remove it (and expose the underlying gold patch) using a selective etchant.

### D. Cantilever geometry design considerations

The optimal cantilevers for biological SMFS experiments are small and soft. Starting with a small cantilever allows for relatively low hydrodynamic drag and fast time response. Small, commercially available cantilevers can have their spring constants lowered to reduce force noise and drift. Beam theory provides a relationship between the spring constant and the dimensions of a cantilever [Eq. (1)], where *E* is the Young’s modulus, *w* is the width, *t* is the thickness, and *L* is the overall length:

$$k_{s}=\frac{E\text{w}t^{3}}{4L^{3}}.$$ (1)

This equation offers a rational approach for designing FIB-milling procedures to achieve desired properties by modifying the width and thickness of the leg(s) of the cantilever. For example, decreasing leg width will decrease the spring constant linearly, whereas decreasing the thickness will decrease *k*_*s*_ cubically. Such arguments were used to design the initial milling strategy for the previously unmodified PeakForce cantilevers (see below).

However, practical considerations prevent the extension of this argument to arbitrarily small and soft cantilevers. First, the starting cantilever must have enough surface area to reflect the detection laser of the AFM. Modification of the cantilever width is constrained by the resolution of the FIB due to focus and lateral sample drift. In addition, a cantilever that is too soft will not survive the transition into liquid necessary for biological experiments. Modifying the cantilever’s thickness causes the cantilever to bend and can only be done to the extent that a horizontal cantilever is maintained, able to properly reflect the AFM detection laser. Thus, a practical milling strategy must be refined through an iterative process to optimize mechanical properties in light of these constraints.

## III. METHODS

### A. BioLever Mini cantilever modification

We adapted published FIB-milling methods^13,16–23,25^ to recreate the desired properties (i.e., lower *k*_*s*_, reduced drift) of modified BioLever Mini cantilevers (Olympus AC40-TS) with a Hitachi NB5000 FIB/SEM instrument, employing an approach with fewer instrument-specific requirements.

Milling time and strategy depend upon the Ga-ion beam conditions of the instrument. The beam conditions of the Hitachi NB5000 are specified in terms of accelerating voltage in kV, current density (denoted 0 for low density and 1 for high density), and beam aperture size in *μ*m. These three conditions are arranged into a three-number combination. For example, a beam with 40 kV accelerating voltage, low-density beam current, and a 30-*μ*m beam aperture size is denoted as 40-0-30. We used condition 40-0-30 for routine Ga-ion imaging, 40-0-80 for all milling and thinning, and 40-1-30 for tungsten deposition. These conditions achieved similar results to prior work specified in terms of accelerating voltage and beam current.^19^

A Hitachi-style ridge mount (Ted Pella, Inc. No. 16356) was used to mount the cantilever tip–down in the FIB [Fig. 1(b)]. The cantilever chip was adhered to the mount using carbon tape on the top of the ridge, making sure that the cantilever itself was suspended off the ridge. The mount was then screwed onto a pin stub. The mounting height was set using a benchtop laser range finder such that the top of the chip was at the instrument eucentric height.

All FIB modifications were monitored by SEM. The FIB beam comes from directly above the sample, the SEM beam is offset by ∼52°, and the tungsten gas nozzle enters from the left [Fig. 1(b)]. FIB and SEM imaging require mechanical translation of the sample stage because the cantilever is not at the eucentric position of the two beams [Fig. 1(c)]. Slight imprecision in motor movement necessitates additional Ga-beam imaging when the cantilever is restored to the FIB position to confirm its precise location. Additional Ga-beam exposure causes bending in the cantilever, and too much exposure can prematurely etch the gold coating. We thus imaged for short lengths of time and used the gentlest feasible beam condition (i.e., 40-0-80).

**FIG. 2.**
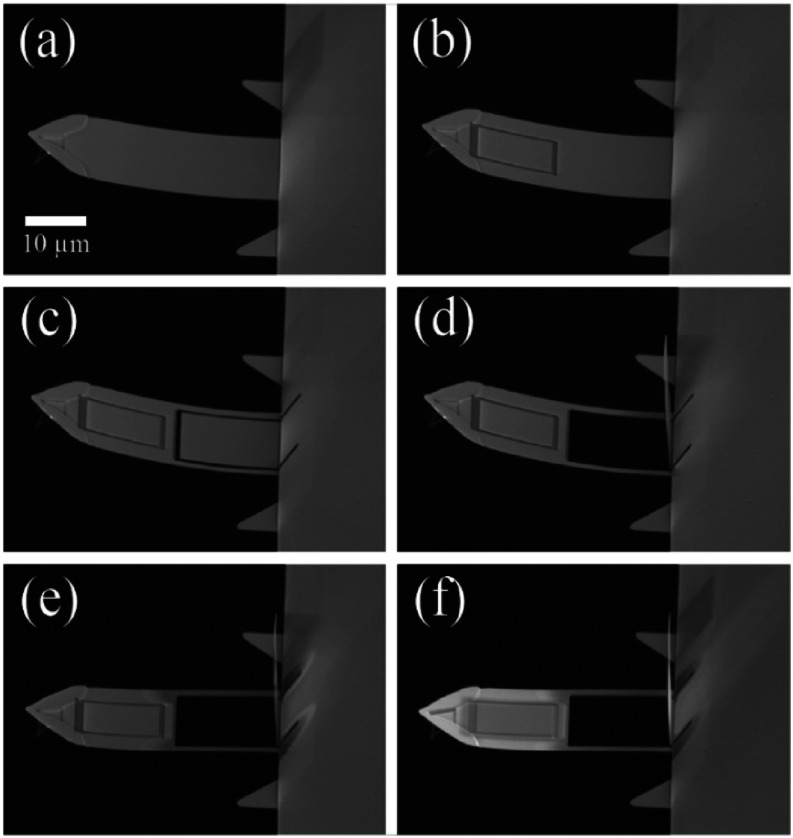
FIB modification of BioLever Mini: (a) Unmodified BioLever Mini cantilever. (b) Patch frame. (c) Leg cuts. (d) Flip up. (e) Leg thinning. (f) Tungsten deposition.

Figure 2 shows the SEM images of the unmodified cantilever [Fig. 2(a)] and the five subsequent modification steps [Figs. 2(b)–2(f)]:

1. The gold and chromium around the tungsten patch area were removed so that the tungsten could adhere to the underlying silicon nitride, ensuring that the tungsten was not undercut during etching. The frame had dimensions of 8 × 12 *μ*m. The sides were created by area cuts; that is, raster scans of the Ga beam in a specified region. Each area cut was 0.5 *μ*m in width and had a specified depth of 0.1 *μ*m to avoid cutting all the way through the cantilever [Fig. 2(b)].
2. Three cuts were made to form two legs that are each 1.5 *μ*m wide [Fig. 2(c)]. These area cuts had a width of 0.5 *μ*m and a depth specified at 0.3 *μ*m to ensure complete cut-through.
3. After cutting the legs, the rectangle between them was flipped up into a vertical orientation by making a sputtering vector cut (i.e., back-and-forth scan along a single line) across the rectangular region near where it meets the chip for 0.7 min [Fig. 2(d)]. This section was flipped up to ensure it did not interact with the legs.
4. We used a defocused sputtering vector cut along the length of both legs to thin them in elevation. This thinning both reduces *k*_*s*_ and corrects for the upward bend of the cantilever induced by Ga-ion imaging. Interestingly, an area cut applied similarly to the legs caused the cantilever to bend up even more. The beam was defocused by 0.15 keV, and the legs thinned for ∼1 min [Fig. 2(e)].
5. A tungsten resist was deposited to facilitate chemical etching (see below). Here, a 40-1-30 beam condition was used. The deposition area was specified to be 9 × 13 *μ*m (enough to cover both the patch and the trenches), and deposition proceeded for 2 min [Fig. 2(f)].

A more detailed step-by-step milling procedure is provided as a supplementary method in the supplementary material.

### B. PeakForce cantilever modification

**FIG. 3.**
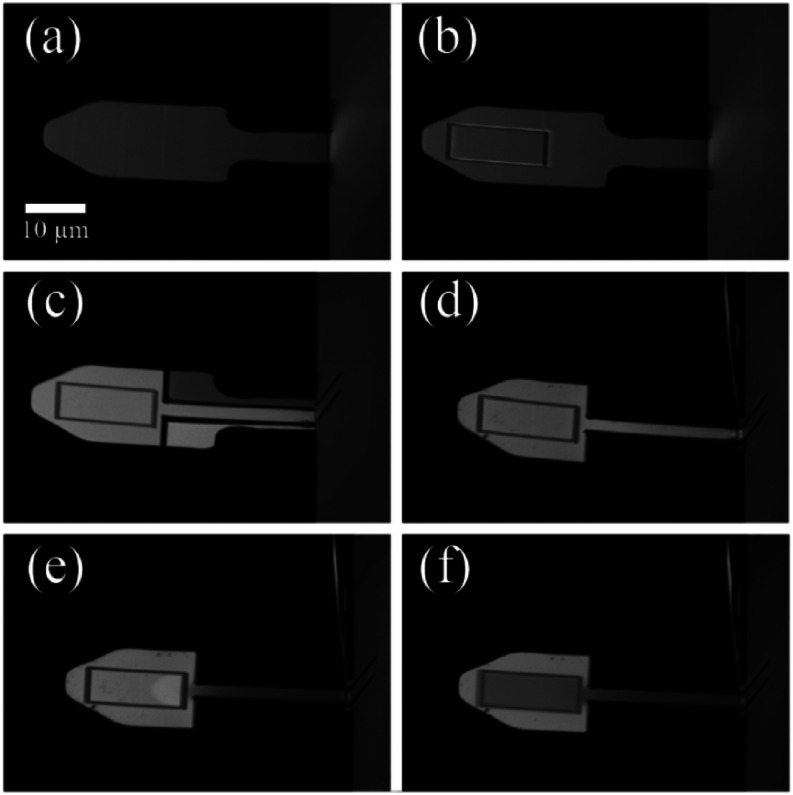
FIB modification of PeakForce cantilever: (a) Unmodified PeakForce cantilever. (b) Patch frame. (c) Leg cuts. (d) Flip up. (e) Leg thinning. (f) Tungsten deposition.

We applied these more general methods to a commercially available cantilever (Bruker PEAKFORCE-HIRS-F-B) not previously subjected to FIB modification. Figure 3 shows SEM images of the unmodified cantilever [Fig. 3(a)] and all subsequent modification steps [Figs. 3(b)–3(f)]. The narrower and thicker leg of the PeakForce cantilever required additional changes in the modification process. Here, after preparing the tungsten frame as before [Fig. 3(b)], four area cuts were made to narrow the leg from 6.8 to 1.5 *μ*m and shorten the head by 7 *μ*m [Fig. 3(c)]. The cuts had a width of 0.5 *μ*m, and the specified depth was 0.5 *μ*m. These cuts were made in two stages, with the long cuts extending to the chip made before the short cuts to the side of the cantilever. We found that this sequence prevented the sides from folding toward the leg. The two side wings were then flipped up using a sputtering vector cut near and parallel to the chip edge for 0.7 min [Fig. 3(d)]. A defocused vector cut was again used to correct for the slight upward tilt of the cantilever induced by Ga-ion imaging and to reduce *k*_*s*_. Here, thinning occurred for 3 min [Fig. 3(e)]. There were no changes in the final tungsten deposition step [Fig. 3(f)].

### C. Chemical etching of gold, chromium, and tungsten

Chemical etchants were used to completely remove metal layers and expose the silicon nitride bulk of the cantilever, except within the tungsten-protected reflective patch. Cantilevers were sequentially treated with gold (Transene Gold Etch TFA, 20 s), chromium (Transene Chromium Etch 1020, 20 s), and tungsten (Sigma-Aldrich Cat. No. 667498, 30 s) etchants by submersion and swirling. Each cantilever was rinsed for 30 s each in isopropanol and ultrapure water after exposure to each etchant and was blotted dry at the end of the process.

### D. Spring-constant calibration and mechanical characterization

Each cantilever’s spring constant was calibrated by first obtaining the deflection inverse optical lever sensitivity (invOLS). The deflection invOLS was measured by pushing into the surface until reaching 1 V in Tris-buffered saline solution. We then calculated the power spectral density (PSD) of thermal fluctuations, which we fit with a model function to obtain *k*_*s*_.^26^ To characterize time response, the autocorrelation was calculated as the inverse Fourier transform of the PSD; the time response was taken as the time in which the autocorrelation decayed to 1/*e*.

## IV. RESULTS AND DISCUSSION

### A. FIB milling of AFM cantilever generalized for platform independence

SMFS data quality and the ability to measure rapid single-molecule conformational changes depend on cantilever mechanical properties, motivating prior FIB-modification work. For example, when applied to BioLever Mini cantilevers (*L* = 38 *μ*m), these procedures have achieved an 8- to 12-fold reduction in spring constant, sub-pN force stability to 80 s, and only a modest increase in response time from 8 to 20 *μ*s.^13^ However, there are challenges in implementing these methods on a broader array of FIB instruments, as described above. Here, we show that we can reproduce the desirable reductions in *k*_*s*_ and drift (while maintaining relatively fast *τ*) using a platform-independent procedure.

As in prior methods, we used FIB milling to reduce the spring constant of the cantilever directly by carving out two narrow legs and further thinning them in elevation but now used alternate approaches to account for instrumental limitations in sample loading and focus control (see Sec. III). As an example, the unmodified cantilever shown in Fig. 2(a) had a spring constant of 105.1 pN/nm. Upon modification and etching, the cantilever spring constant was lowered to 9.7 pN/nm. On average, modified cantilevers saw a 10-fold reduction in spring constant (*N* = 4), consistent with previous literature reports of an 8- to 12-fold reduction.^13,18–20^ Further leg narrowing beyond 1.5 *μ*m would be expected to further lower *k*_*s*_, but, for example, 1.2 *μ*m legs were found to lead to high breakage rates when the cantilever was immersed in liquid etchants.

**FIG. 4.**
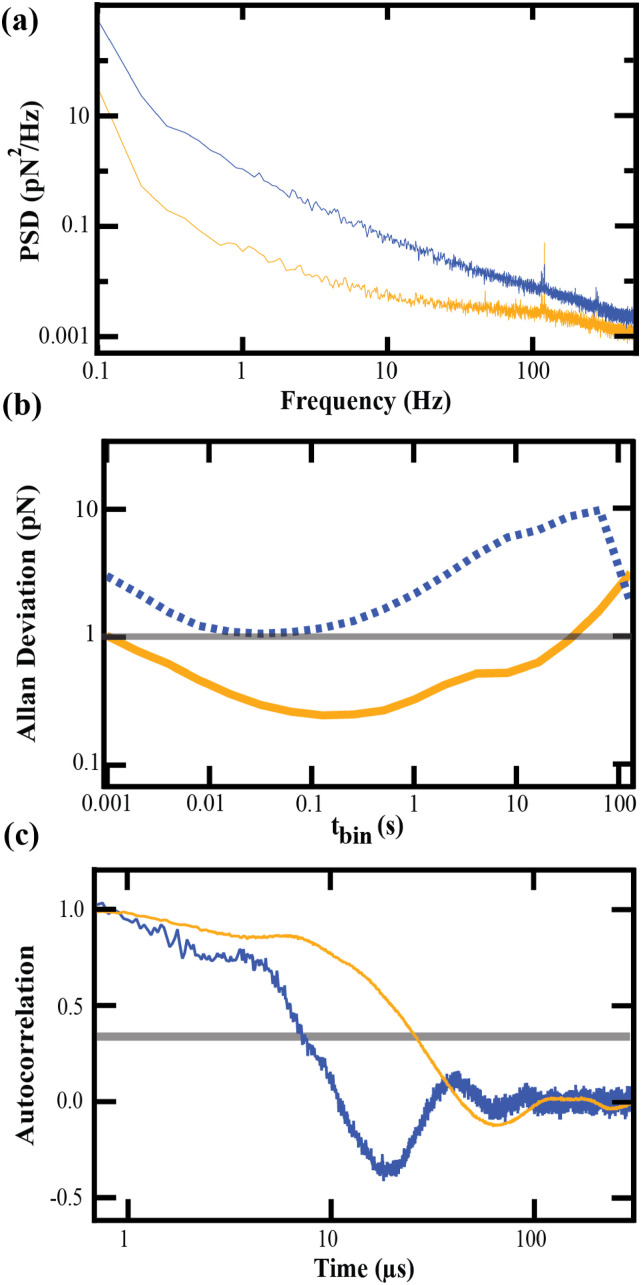
Thermal characterization of unmodified (*blue*) and modified (*orange*) BioLever Mini cantilevers: (a) Power spectral density. (b) Allan deviation, a representation of force precision; the horizontal gray line represents the region of sub-pN force stability. (c) Autocorrelation. The horizontal gray line denotes 1/*e*; the time of crossing this threshold represents the time response *τ* of the cantilever.

A concurrent reduction in drift—attributed in prior literature to reducing *k*_*s*_ and to etching—is quantified through measurements of the thermal motion of the freely vibrating cantilever. The PSD of this motion [Fig. 4(a)] shows an overall decrease across all frequencies, indicating the expected reduction in force noise with *k*_*s*_, and an especially pronounced reduction at low frequencies, corresponding to a decrease in drift. Another way to visualize these effects is to examine the Allan deviation, which expresses force precision over different time scales by calculating the variance of data averaged over different bin sizes (*t*_bin_). The Allan deviation plotted in Fig. 4(b) is calculated from 1 kHz data and spans averaging windows from milliseconds to hundreds of seconds (5 MHz Allan deviation data are shown in Fig. S1 of the supplementary material). Allan deviation plots allow readout of the timescales over which force precision exceeds a desired threshold; for example, the timescale of sub-pN force stability, a reasonably small level of drift for SMFS studies. The unmodified cantilever in Fig. 4(b) shows force stability above 1 pN on all timescales, whereas the modified cantilever exhibits stability below 1 pN up to 50 s. This is comparable to the 50–100 s of sub-pN stability seen previously.^13,16^

Despite a 90% reduction in the spring constant, time response was maintained below 50 *μ*s. As in prior literature, we quantified time response as the timescale of exponential decay in the autocorrelation of cantilever thermal motion, setting *τ* equal to the time needed for the autocorrelation to decay to 1/*e* [gray line, Fig. 4(c)]. Modification shifts the time response to higher times, a necessary trade-off in decreasing *k*_*s*_. Prior modification of BioLever Mini cantilevers was accompanied by a ∼3-fold increase in *τ*, from 6 to 15 *μ*s.^13,19^ Our modified procedures produced a similar result, with *τ* increasing from 9.6 to 25.2 *μ*s upon modification. The response of our modified cantilever to molecular attachment, like that experienced in an SMFS experiment, was additionally measured in an assay of the cantilever’s nonspecific adhesion to a surface-adhered biomolecule. The details of this assay are given as supplementary methods in the supplementary material. Sampling of the final detachment of the cantilever from its surface tether at 5 MHz allowed us to directly observe the relaxation of the cantilever to its equilibrium position (Fig. S2 of the supplementary material). This relaxation was fit with an exponential function having *τ* = 21.6 *μ*s. This value is similar to that estimated from the autocorrelation analysis, confirming that the response times obtained from that method reflect the relaxation of the cantilever in an SMFS experiment.

### B. Extension of FIB milling to an alternate, commercially available cantilever

Whereas the BioLever Mini cantilever discussed above and in prior literature has been discontinued by the manufacturer, these generalized methods can be extended to presently commercially available cantilevers. As an example, we used FIB modification to tune the mechanical properties of the Bruker PEAKFORCE-HIRS-F-B cantilever to achieve performance similar to the BioLever Mini. The PeakForce cantilever [Fig. 3(a), *L* = 36 *μ*m] already has a single narrow leg (5 *μ*m width, compared to the 16 *μ*m width of the BioLever Mini), but it is thicker in elevation than the BioLever Mini (250 nm vs 200 nm), leading to a similar unmodified spring constant (*k*_*s*_ = 120 pN/nm vs 100 pN/nm for the BioLever Mini). The competing effects of leg width and thickness are understood in terms of beam theory [Eq. (1)]. Because the PeakForce already has a single leg, we chose to narrow this one leg rather than cut it into two. We needed to narrow the leg to at most the combined width of the two modified BioLever Mini legs (∼2.4 *μ*m) and, in fact, needed to further narrow it (∼1.5 *μ*m) to account for the inability to thin the leg in elevation as much as those of the BioLever Mini. This is because leg thinning also bends the cantilever, which must be kept horizontal for AFM detection. A modification of the BioLever Mini procedure, likewise applicable to a broad range of FIB instruments but now involving altered geometry and milling times, achieved comparable mechanical properties with the PeakForce cantilever (see Sec. III).

**FIG. 5.**
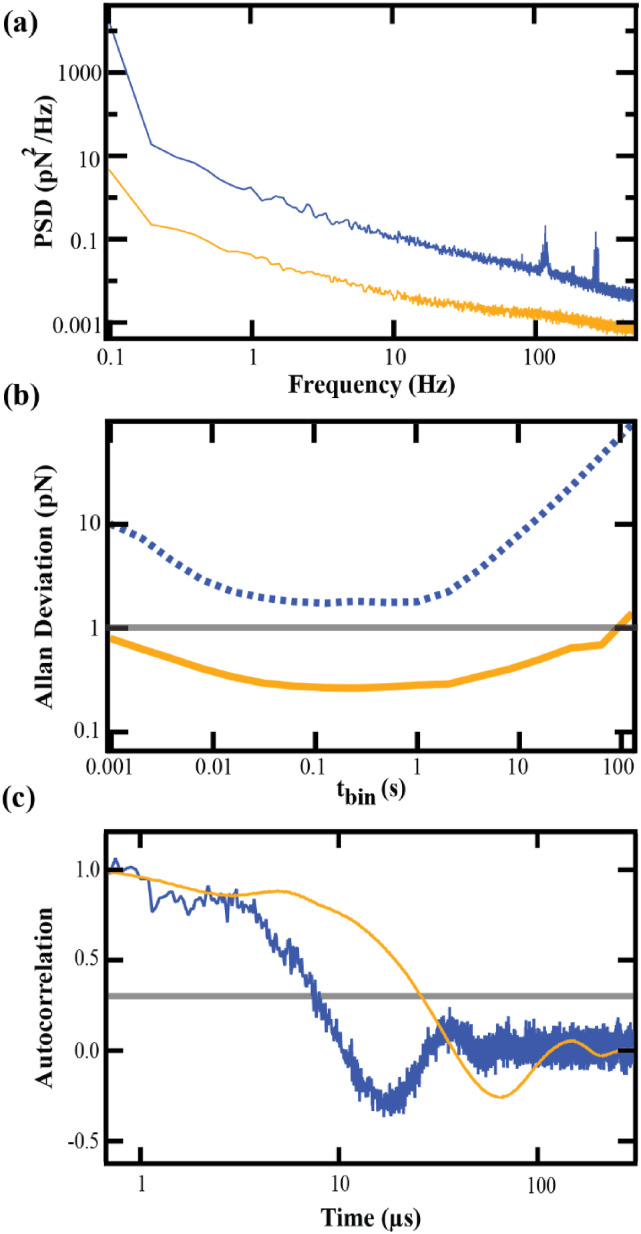
Thermal characterization of unmodified (*blue*) and modified (*orange*) PeakForce cantilevers: (a) Power spectral density. (b) Allan deviation, a representation of the force precision; the horizontal gray line represents the region of sub-pN force stability. (c) Autocorrelation. the horizontal gray line denotes 1/*e*; the time of crossing this threshold represents the time response *τ* of the cantilever.

Figure 5 gives an example of these methods applied to a PeakForce cantilever with unmodified *k*_*s*_ = 121.9 pN/nm. Upon FIB milling and etching, this cantilever exhibited a spring constant of 12.9 pN/nm. On average, PeakForce cantilevers modified in this way showed an 88% reduction in *k*_*s*_ (*N* = 3), comparable to the 90% reduction seen for the BioLever Mini.

The PSD [Fig. 5(a)] shows an overall decrease in noise and drift, similar to the BioLever Mini. The Allan deviation [Fig. 5(b)] of the 1 kHz force–noise data shows that the unmodified PeakForce does not fall below 1 pN over any time window, whereas the modified cantilever exhibits sub-pN stability up to 20 s (see Fig. S3 of the supplementary material for 5 MHz Allan deviation). Even though the window of low-drift stability is slightly smaller for the PeakForce, it still spans about three decades from ∼0.02 to ∼20 s and is sufficient to collect many seconds of SMFS data on individual molecules.

The modified PeakForce also shows a time response comparable to the BioLever Mini. The time constant derived from autocorrelation analysis of PeakForce cantilevers [Fig. 5(c)] shifts from 7 to 25 *μ*s upon modification, a ∼3-fold increase consistent with BioLever Mini results in both our treatment and that of prior literature.

## V. CONCLUSION

In this work, we have shown that modified techniques, applicable to a broader range of FIB instrumentation, can reproduce previously reported cantilever performance improvements for the Olympus AC40TS cantilever and can be extended to a presently commercially available cantilever. We demonstrated the success of these modified techniques through mechanical and noise characterization in the liquid environment relevant to biological measurements.

There are costs associated with this cantilever-modification strategy. For the designs considered here, it took about 3 h of FIB equipment time to modify four cantilevers. At research universities in our area (southeastern United States), FIB usage costs about $80/h. Thus, the modification costs about $60/cantilever. This is compared to the base cost of the unmodified cantilevers: about $90/cantilever in the case of the PEAKFORCE-HIRS-F-B. In addition, cantilevers with significantly softer spring constants are more susceptible to damage, especially due to hydrodynamic forces when passed from air to liquid. Depending on handling conditions, this may limit the number of times the cantilever can be repeatedly reused in experiments.

Despite these costs, however, FIB-modified cantilevers can achieve a favorable noise, drift, and time-response profile not found in commercially available cantilevers. The methods reported here raise the prospect of more widespread adoption of modified cantilevers in biological SMFS, enabling finer resolution of protein unfolding pathways and observation of rapid, reversible transitions amenable to equilibrium energetic analysis. In such an application, site-specific attachment chemistry can be used to chemically functionalize the modified cantilevers.^27^ They can then be raster scanned above a surface, repeatedly being lowered to make contact and form attachments with molecules either attached to the surface via linkers (in the case of soluble proteins) or embedded in a supported lipid bilayer (in the case of membrane proteins). The protein can then be unfolded at constant velocity to reveal its characteristic force-extension curve reflecting an ensemble of unfolding intermediates, unfolded at variable pulling speeds to enable dynamic force spectroscopy to reveal transition-state information, or held under constant tension to access equilibrium thermodynamics.^28^ To fully leverage the high time resolution of the cantilever, data should be collected at high bandwidth and then downsampled by smoothing to *τ*.

## SUPPLEMENTARY MATERIAL

Supplementary material includes additional figures (Figs. S1–S3), a table (Table S1), and supplementary methods. These methods include a more detailed, step-by-step description of the FIB-milling procedure and the experimental conditions used to generate Fig. S2.

## Data Availability

The data that support the findings of this study are available from the corresponding author upon reasonable request.
